# Sexual and reproductive health self-care in humanitarian and fragile settings: where should we start?

**DOI:** 10.1186/s13031-021-00358-5

**Published:** 2021-04-07

**Authors:** Nguyen Toan Tran, Hannah Tappis, Pierre Moon, Megan Christofield, Angela Dawson

**Affiliations:** 1 Australian Centre for Public and Population Health Research, Faculty of Health, University of Technology Sydney, PO Box 123, Sydney, NSW 2007 Australia; 2 Faculty of Medicine, University of Geneva, Rue Michel Servet 1, 1211 Geneva 4, Switzerland; 3 Johns Hopkins Center for Humanitarian Health, 615 N. Wolfe St, Baltimore, MD USA; 4 Population Services International, 1120 19th St. NW, Suite 600, Washington, DC, 20036 USA; 5 Self-Care Trailblazer Group, 1120 19th St. NW, Suite 600, Washington, DC, 20036 USA

**Keywords:** Self-care, Sexual and reproductive health, Humanitarian settings, Fragile settings, Minimum initial service package (MISP), Interventions, Program, Policy, Research

## Abstract

Recent crises have accelerated global interest in self-care interventions. This debate paper aims to raise the issue of sexual and reproductive health (SRH) self-care and invites members of the global community operating in crisis-affected settings to look at potential avenues in mainstreaming SRH self-care interventions. We start by exploring self-care interventions that could align with well-established humanitarian standards, such as the *Minimum Initial Service Package (MISP) for Sexual and Reproductive Health in Crises*, point to the potential of digital health support for SRH self-care in crisis-affected settings, and discuss related policy, programmatic, and research considerations. These considerations underscore the importance of self-care as part of the care continuum and within a whole-system approach. Equally critical is the need for self-care in crisis-affected settings to complement other live-saving SRH interventions—it does not eliminate the need for provider-led services in health facilities. Further research on SRH self-care interventions focusing distinctively on humanitarian and fragile settings is needed to inform context-specific policies and practice guidance.

## Background

### Sexual and reproductive health self-care

Self-care for sexual and reproductive health (SRH) has equipped people, especially women and girls, with skills and knowledge passed through generations to manage menstruation, fertility, pregnancy, and childbirth for themselves and care for their newborns and children [1]. According to the World Health Organization (WHO), “*Self-care is the ability of individuals, families and communities to promote health, prevent disease, maintain health, and cope with illness and disability with or without the support of a health care provider*” [2]. Such self-directed health is increasingly valued as a critical asset of the healthcare ecosystem with great potential to fill SRH service gaps and bolster universal health coverage when situated within a rights-based, gender-sensitive, people-centered, and health-system integrated approach [3, 4]. Such interventions may save money for both users and the healthcare system through a mixed financing model that includes public and private sector financing and direct user payment [5]. SRH self-care interventions could play a larger role in humanitarian settings, where trained health workers and adequate health infrastructure are lacking [6]. Recent systematic reviews were published in 2019 and show the promise of self-injected contraceptives, over-the-counter oral contraceptive pills, home-based ovulation predictor kits, self-sampling for human papillomavirus, and self-testing for sexually transmitted infections [7–11]. Most of the identified studies were done in high-income countries and none in humanitarian settings.

### Fragile and humanitarian settings

Approximately 1.8 billion people live in fragile settings around the world, including 168 million in humanitarian contexts [12]. Around a quarter are women and girls of reproductive age [13]. SRH conditions are among the principal causes of death and ill-health among women of childbearing age worldwide, with 61% of maternal deaths occurring in countries experiencing fragility and crisis [14]. While the prioritization and coverage of SRH services in humanitarian settings have expanded over the last few decades, there continue to be significant unmet needs [15]. Funding for humanitarian assistance is limited, and variations in socio-political contexts, health system capacity, population movement, security, and humanitarian access challenge the provision and utilization of essential health services in many settings [16]. For many fragile and crisis-affected countries, natural or human-made disasters represent an additional and unparalleled burden to already overwhelmed health systems, with significant implications for women and girls, but also men and boys [17, 18]. As exemplified by the coronavirus disease 2019 (COVID-19) pandemic, home confinement and travel restrictions combined with the fear of contracting the disease and the closure or limited hours of healthcare facilities and stock-outs of essential medications and equipment have delayed the uptake of and timely access to essential health services with a gendered impact on women and girls, thus rendering gender-inclusive—and age, disability, and diversity-inclusive—leadership and strategies even more relevant [18–21]. Previous experience of epidemics in fragile and humanitarian settings indicates that the interruption of healthcare services considered unrelated to the epidemic response might have occasioned more deaths, including SRH-related deaths, than did the epidemic itself [22, 23].

### Debate rationale

Against this background, we believe that the time is ripe for programs in fragile and humanitarian settings to consider systematically implementing SRH self-care interventions. However, based on our field knowledge, programmatic models to assist decision-makers in allocating resources are lacking to guide the safe and effective implementation of SRH self-care interventions in settings with often severely disrupted health systems. This debate paper does not propose such programmatic models, nor does it offer a systematic review of existing guidance and practices on the topic—none of the systematic reviews recently published and mentioned in the introduction retrieved studies done in fragile or humanitarian settings. Instead, our debate paper aims to raise the issue among the members of the global SRH community operating in crisis-affected settings and invites them to explore together potential avenues in mainstreaming SRH self-care interventions. Consequently, the paper starts with an exploration of self-care interventions that could align with well-established humanitarian standards, touches upon the potential of digital health support for SRH self-care in crisis-affected settings, and discusses related policy, programmatic, and research considerations. Our reflection acknowledges that the programmatic implications for including self-care interventions will vary vastly depending on the setting, intervention type, where and how they are accessed, and the required links to the health system to support care.

## Intersections of self-care with SRH in crisis settings

Individuals living in humanitarian or fragile settings may increasingly resort to SRH self-care, as crises may accelerate the inequities in access to healthcare providers and services. A more deliberate application of self-care that recognizes underlying inequities and seeks to mitigate, rather than exploit them, could be particularly appropriate for increasing health coverage. The WHO self-care guideline includes a good practice statement (GPS 8) recommending that the “*Provision of tailored and timely support for self-care interventions, including for SRHR [SRH and rights], in humanitarian settings should be in accordance with international guidance, form part of emergency preparedness plans and be provided as part of ongoing responses*” [24].

The Sphere Handbook recognizes that the Minimum Initial Service Package (MISP) for SRH is the global standard in humanitarian response [25]. Four MISP objectives address the provision of SRH services that aim to save lives or alleviate diseases and suffering during the initial phase of an emergency: prevent sexual violence and respond to the needs of survivors (Objective 2); prevent the transmission of and reduce morbidity and mortality due to HIV and other sexually transmitted infections (Objective 3); prevent excess maternal and newborn morbidity and mortality (Objective 4); and prevent unintended pregnancies (Objective 5) --as another priority, safe abortion care should be available to the full extent of the law. Meanwhile, two objectives focus on coordination and planning: ensuring the health sector/cluster identifies an organization to lead the implementation of the MISP (Objective 1) and planning for comprehensive SRH services, integrated into primary health care as soon as possible (Objective 6).

Comprehensive SRH services should build upon the MISP and be integrated into primary care whenever possible in a crisis. Comprehensive SRH services include, among others, additional maternal and neonatal health, family planning, HIV, and gender-based violence services not included in the MISP (e.g., antenatal and postnatal care) as well as interventions related to infertility services, cervical cancer, other gynecological morbidities, and sexual health [26].

SRH self-care interventions present opportunities to advance many of the MISP objectives. Table 1 presents self-care interventions that could align with the MISP objectives and activities. The final row addresses Objective 6 of the MISP with additional interventions that emergency and development actors can introduce as part of the restoration of, or transition to, comprehensive SRH during the recovery phase of a crisis. This list is an initial exploration of potential interventions that could feed into our debate and stems from a review of the MISP activities that intersect with interventions recommended in key relevant publications [24, 27]. The listed interventions should be locally contextualized and implemented as part of a continuum of care and within a whole-system approach [28]. Therefore, humanitarian actors are invited to consider and enrich the programmatic, policy, and research considerations we have outlined hereunder.

**Table 1:** Self-care interventions aligned with the MISP for SRH in humanitarian settings

MISP objectives and activities	Self-care interventions that align with the MISP
**1. Coordination:** Ensure the health sector/cluster identifies an organization to lead the implementation of the MISP	Not applicable. However, the implementation of self-care interventions should be the fruit of concerted policy and programmatic efforts at all levels.
**2. Gender-based violence:** Prevent sexual violence and respond to the needs of survivors. *Activities:* • Establish measures to prevent sexual violence • Provide clinical care for survivors of sexual violence (treatment of injuries, post-rape care, mental health and psychosocial support, safety planning, referrals)	• Care of injuries • Over the counter oral contraceptive pills (WHO Rec 11) • Emergency contraception • HIV post-exposure prophylaxis • STI presumptive treatment • Positive coping methods
**3. HIV/STI:** Prevent the transmission and reduce morbidity and mortality due to HIV and other STIs. *Activities*: • Safe and rational blood transfusion • Ensure standard precautions • Provide condoms • Continue treatment for people enrolled in antiretroviral therapy (ART), including women enrolled in PMTCT • Provide post-exposure prophylaxis (PEP) for survivors of sexual violence • Provide cotrimoxazole prophylaxis for opportunistic infections for patients already diagnosed with HIV	• Condom use (WHO Rec 12–13) • HIV post-exposure prophylaxis for survivors of sexual violence • ART treatment, for people already enrolled including pregnant and postpartum women • Cotrimoxazole prophylaxis
**4. Maternal and newborn health:** Prevent excess maternal and newborn morbidity and mortality. *Activities*: • Safe and clean delivery • Essential newborn care • Provide emergency obstetric and newborn care • Provide post-abortion care	• Misoprostol for prevention of postpartum hemorrhage • Chlorhexidine for neonatal cord care • Other components essential newborn care (thermal care, breastfeeding, etc.) • Post-abortion hormonal contraception initiation (WHO Rec 19–20)
**5. Contraception**: Prevent unintended pregnancies. *Activities*: • Ensure a range of long-acting and short-acting contraceptive methods • Provide information and ensure awareness of the availability of contraceptives	• Self-administration of injectable contraception (WHO Rec 10) • Over the counter oral contraceptive pills (WHO Rec 11) including emergency contraception • Up to 1-year supply oral contraceptive pills (WHO Rec 15) • Condom use (WHO Rec 12–13) • Post-abortion hormonal contraception initiation (WHO Rec 19–20) • Postpartum contraception initiation • Lactational amenorrhea method • Fertility awareness-based / standard day methods• Traditional methods (e.g., withdrawal)
***Other priority:*** **Safe abortion care.** Ensure safe abortion care is available in health centers and hospitals, to the full extent of the law	• Self-management of medical abortion process in the first trimester (WHO Rec 16–18) • Post-abortion hormonal contraception initiation (WHO Rec 19–20)
**6. Transition to comprehensive SRH:** Plan for comprehensive SRH services, integrated into primary care as soon as possible. *Activities*: • Work with the health sector/cluster to address the six health system building blocks	**SRH Self-Care Interventions beyond the MISP** • HIV self-testing (WHO Rec 23) • ART programming for new enrollees • HIV pre-exposure prophylaxis (oral PrEP) • Self-collection of samples for STI testing (WHO Rec 22) • Self-administered pain relief for prevention of delay in the first stage of labor (WHO Rec 9) • Self-administered interventions for common physiological symptoms of pregnancy (WHO Rec 3–8) • Non-clinical interventions to reduce unnecessary cesarean sections (WHO Rec 1 & 2) • Cancer: Self-sampling for HPV testing (WHO Rec 21), breast cancer self-exam, testicular self-exam • Fertility: Home-based ovulation predictor kits (WHO Rec 11), home-based pregnancy tests, menstrual health management • Sexual health: Sexuality education • Mental health: Positive coping, self-help

Where relevant, the recommendation numbers from the WHO guideline on SRH self-care are reported for ease of reference (“WHO Rec + number”) [24]. The listed interventions in the right column should be locally contextualized and implemented as part of a continuum of care within a whole-system approach

The *WHO Consolidated Guideline on Self-Care Interventions for Health: Sexual and Reproductive Health and Rights (2019)* provides the latest recommendations on self-care [24]. This guidance is meant to support individuals, communities, and countries to develop people-centered, quality health services and self-care interventions based on systematic evidence reviews. The quality and certainty of the currently available evidence supporting these interventions were assessed according to WHO procedures for guideline development. Interventions from this guideline included in Table 1 are accompanied by the related recommendation number for ease of reference.

Other interventions included in Table 1 have not yet gathered sufficient evidence, although this does not speak against their potential role in self-care. These interventions stem from the MISP and the more comprehensive *Inter-Agency Field Manual on Reproductive Health in Humanitarian Settings* (2018), which was reviewed by WHO prior to publication [29]. To expand our exploration, we also examined the WHO publication on *Packages of Interventions for Family Planning, Safe Abortion Care, Maternal, Newborn and Child Health* (2010) for other self-care interventions as it offers a valuable overview of the care continuum in the life course starting at the home/community level, in addition to first-level health facilities and referral facilities [27]. This tentative list can be useful as a launchpad for a rigorous review of the literature on interventions, opportunities, and gaps related to SRH self-care in humanitarian settings (a scoping review started in December 2020 [30]).

## The enabling potential of digital health for SRH self-care in crisis settings

While digital health does not yet feature as a component of the MISP, COVID-19 has dramatically accelerated the role of digital technologies in healthcare, with substantial implications for self-care and healthcare, from the rapid expansion of telemedicine to smartphone applications for enhanced self-diagnosis and treatment management [31]. Due to the growing number of fragile and humanitarian settings or neighboring host countries with reliable mobile phone ownership and connectivity, we foresee that the use of mobile phones and other digital health technologies could offer a promising platform to explore and advance the implementation of SRH self-care in these settings [32]. Remote client-provider consultations or dispensing instructions or support for self-care through WhatsApp or Facebook discussion groups could expand SRH services. For example, Syrian adolescent girls and young women who found asylum in Turkey reported widespread ownership of cellular phones, which enabled them to access gender-based violence interventions through a mobile platform [33].

If digital health is to advance equitable, affordable, quality healthcare, especially in contexts where men may have disproportionate access to mobile and digital technologies, this new healthcare delivery landscape will have to continuously address many of the same requirements of provider-led and facility-based care. Such requirements include ensuring privacy and confidentiality, avoiding harm from the spread of misinformation, and striving for affordability, accessibility, a user-centered design that considers the health and information literacy skills of individuals as well as other dimensions of quality of care that facilitate positive user experiences and outcomes [34]. The *2019 WHO Guidelines for Digital Development*, which focuses on strengthening systems for health, offers a foundation for humanitarian and SRH actors to build upon as digital capacity is leveraged in support of self-care and healthcare in humanitarian and fragile settings—a trend that will only continue to grow in a post-COVID-19 world [35, 36].

## Policy, program, and research considerations

The different possibilities for advancing self-care interventions within the MISP objectives illustrate the potential for these interventions to expand and maintain SRH access in fragile and disrupted health system contexts. Advancing the role of self-care in the MISP requires a coordinated approach ideally informed by established evidence. With self-care increasingly front-of-mind in health system planning and delivery, health actors in acute and protracted crises could catalyze collective learning as they implement SRH self-care interventions and document lessons learned in settings with varying levels and types of humanitarian disasters. To this end, we have reflected on the following considerations that could help SRH policymakers, program managers, and researchers safeguard self-care interventions in humanitarian and fragile settings.

First, self-care complements other live-saving SRH interventions and does not eliminate the need for provider-led facility-based care. As underscored by the need for a health system approach to self-care, critical and immediately lifesaving services and supplies defined by the MISP should continue due to the high risks of untreated medical complications [28]. For instance, obstetric surgery requires skilled providers operating in health facilities with adequate clinical infrastructure, technologies, and supplies. Comprehensive SRH services, if already in place, should continue as long as the system can cope [37]. As illustrated by the COVID-19 response, remote approaches should be considered where feasible for relevant consultations and follow-up (e.g., telephone, digital applications, text messaging).

Second, self-care for SRH is part of a continuum of care within a whole-system approach [3]. Such a continuum encompasses individuals, their community, vendor outlets, healthcare facilities, and the overall public health system, even where resources are scarce. Hence, the introduction or expansion of self-care interventions as part of MISP implementation or more comprehensive SRH services should be the fruit of concerted policy and programmatic efforts at all levels. Such efforts should meaningfully engage community-based organizations and groups representing vulnerable populations and be informed by specific actions required during the different phases of the emergency management cycle, from mitigation and preparedness to response and recovery [38].

Third, there is a need for research on SRH self-care interventions in humanitarian settings. Further evidence and programmatic guidance are needed to support the implementation of WHO good practice statement in diverse humanitarian settings. The *WHO consolidated guideline on self-care interventions* referenced in Table 1 is informed by evidence that does not extensively address programmatic or contextual considerations in humanitarian settings [24]. Further research is required to understand the knowledge, attitudes, and practices of individuals and communities towards self-care in humanitarian settings and provide insights into the social, cultural, legal, political, economic, and health system factors and resources that SRH self-care initiatives could harness [34]. For example, self-care initiatives provide the option of de-medicalized person-centered approaches to healthcare that affect the balance of power perpetuated by the biomedical model of health professionals as experts. In some contexts, laws may facilitate or limit self-care interventions. There are also opportunities to assess the impact self-care may have on current services, such as reduced dependency on and complementarity to facility-based or provider-initiated care. Understanding how the mass media, social media, and other existing communication channels in crisis-affected settings can be mobilized to promote and support self-care initiatives is equally important.

Finally, evidence of the effectiveness of self-care interventions is needed to appreciate the extent to which they are of quality, accountable to people—determining who should hold that accountability is equally important—and can protect them from medical and social harm, violence, coercion, and discrimination. Conversely, knowledge is required to understand if such practices reinforce unsafe and harmful self-medicalization in humanitarian and fragile settings.

## Conclusions

Preparedness and response measures to COVID-19 and other potential humanitarian crises have accelerated global interest in self-care interventions. Although global guidelines on SRH services in humanitarian settings do not explicitly address self-care as a mode for their delivery, some of the highlighted interventions have already been established or are emerging within humanitarian health programming. The potential of self-care to be facilitated through digital means further positions it for achieving greater health coverage and achieving the clinical objectives of the MISP in these settings. Self-care is rooted in individual choice and agency and should be a foundation to optimize the efficiency of facility-based and provider-led care while emboldening people’s participation in their health.

Promoting safe and appropriate self-care interventions requires a sound assessment of user-profiles and preferences, existing self-care practices, and venues and means where self-care instructions, interventions, and products can be accessed. An understanding of the contextual factors that can hinder or enable the effective implementation of self-care during the mitigation, preparedness, response, and response phases of an emergency is needed. The perspectives expressed in this exploratory paper are limited by the diversity and complexities of fragile and humanitarian contexts, combined with the breadth and depth of self-care as an approach to healthcare. Nonetheless, we hope that the preliminary list of MISP-aligned SRH self-care interventions could assist members of the global SRH community working in crisis-affected situations to brainstorm on the design of novel service delivery strategies and use of technology that are supported by appropriate programming, policy, and research considerations.

Ultimately, individuals living in humanitarian and fragile settings must be empowered to practice evidence-informed self-care. Enabling people to identify their own health needs and access appropriate information and interventions to prevent adverse health issues and manage their health conditions promotes self-reliance and autonomy. These actions, in turn, place individuals at the center of a health system, which is vital for all contexts, but especially for strained health systems in fragile and humanitarian settings.

## Data Availability

Not applicable.

## Abbreviations

ART: Antiretroviral therapy
GPS: Good practice statement
HIV: Human Immunodeficiency Virus
MISP: Minimum Initial Services Package for Sexual and Reproductive Health in Crises
PEP: Post-exposure prophylaxis
SRH: Sexual and reproductive health
STI: Sexually transmitted infections
WHO: World Health Organization

## References

[1] Hardon A, Pell C, Taqueban E, Narasimhan M. Sexual and reproductive self care among women and girls: insights from ethnographic studies. BMJ. 2019;365:l1333. 10.1136/bmj.l1333. 10.1136/bmj.l1333 PMC6441865 30936060

[2] World Health Organization (2013). Self care for health: a handbook for community health workers & volunteers.

[3] Narasimhan M, de Iongh A, Askew I, Simpson P J. It’s time to recognise self care as an integral component of health systems. BMJ. 2019;365:l1403.10.1136/bmj.l1403. 10.1136/bmj.l1403 PMC6442398 30936068

[4] Narasimhan M, Logie CH, Gauntley A, Gomez Ponce de Leon R, Gholbzouri K, Siegfried N, Abela H, Ouedraogo L (2020). Self-care interventions for sexual and reproductive health and rights for advancing universal health coverage. Sex Reprod Health Matters.

[5] Remme M, Narasimhan M, Wilson D, Ali M, Vijayasingham L, Ghani F, Allotey P (2019). Self care interventions for sexual and reproductive health and rights: costs, benefits, and financing. BMJ (Clinical research ed).

[6] Logie CH, Khoshnood K, Okumu M, Rashid SF, Senova F, Meghari H, Kipenda CU (2019). Self care interventions for sexual and reproductive health: self care interventions could advance sexual and reproductive health in humanitarian settings. BMJ (Clinical research ed).

[7] Kennedy CE, Yeh PT, Gaffield ML, Brady M, Narasimhan M (2019). Self-administration of injectable contraception: a systematic review and meta-analysis. BMJ Glob Health.

[8] Kennedy CE, Yeh PT, Gonsalves L, Jafri H, Gaffield ME, Kiarie J, Narasimhan ML (2019). Should oral contraceptive pills be available without a prescription? A systematic review of over-the-counter and pharmacy access availability. BMJ Glob Health.

[9] Yeh PT, Kennedy CE, Van der Poel S, Matsaseng T, Bernard L, Narasimhan M (2019). Should home-based ovulation predictor kits be offered as an additional approach for fertility management for women and couples desiring pregnancy? A systematic review and meta-analysis. BMJ Glob Health.

[10] Yeh PT, Kennedy CE, De Vuyst H, Narasimhan M (2019). Self-sampling for human papillomavirus (HPV) testing: a systematic review and meta-analysis. BMJ Glob Health.

[11] Ogale Y, Yeh PT, Kennedy CE, Toskin I, Narasimhan M (2019). Self-collection of samples as an additional approach to deliver testing services for sexually transmitted infections: a systematic review and meta-analysis. BMJ Glob Health.

[12] Organisation for Economic Co-operation and Development (2018). States of fragility 2018.

[13] United Nations Office for the Coordination of Humanitarian Affairs (2020). Global humanitarian overview.

[14] World Health Organization (2017). Maternal mortality: levels and trends 2000–2017; estimates from WHO, UNICEF, UNFPA, World Bank Group and the United Nations Population Division.

[15] Singh NS, Smith J, Aryasinghe S, Khosla R, Say L, Blanchet K (2018). Evaluating the effectiveness of sexual and reproductive health services during humanitarian crises: a systematic review. PLoS One.

[16] Singh NS, Aryasinghe S, Smith J, Khosla R, Say L, Blanchet K (2018). A long way to go: a systematic review to assess the utilisation of sexual and reproductive health services during humanitarian crises. BMJ Glob Health.

[17] Poole DN, Escudero DJ, Gostin LO, Leblang D, Talbot EA (2020). Responding to the COVID-19 pandemic in complex humanitarian crises. Int J Equity Health.

[18] Fuhrman S, Kalyanpur A, Friedman S, Tran NT (2020). Gendered implications of the COVID-19 pandemic for policies and programmes in humanitarian settings. BMJ Glob Health.

[19] Lazzerini M, Barbi E, Apicella A, Marchetti F, Cardinale F, Trobia G (2020). Delayed access or provision of care in Italy resulting from fear of COVID-19. Lancet Child Adolesc Health.

[20] Meagher K, Singh NS, Patel P (2020). The role of gender inclusive leadership during the COVID-19 pandemic to support vulnerable populations in conflict settings. BMJ Glob Health.

[21] Lokot M, Avakyan Y (2020). Intersectionality as a lens to the COVID-19 pandemic: implications for sexual and reproductive health in development and humanitarian contexts. Sex Reprod Health Matters.

[22] Sochas L, Channon AA, Nam S (2017). Counting indirect crisis-related deaths in the context of a low-resilience health system: the case of maternal and neonatal health during the Ebola epidemic in Sierra Leone. Health Policy Plann.

[23] McQuilkin PA, Udhayashankar K, Niescierenko M, Maranda L (2017). Health-care access during the Ebola virus epidemic in Liberia. Am J Trop Med Hyg.

[24] World Health Organization (2019). WHO consolidated guideline on self-care interventions for health: sexual and reproductive health and rights.

[25] Sphere Project (2018). Sexual and reproductive health in sphere handbook - humanitarian charter and minimum standards in humanitarian response.

[26] Starrs AM, Ezeh AC, Barker G, Basu A, Bertrand JT, Blum R, Coll-Seck AM, Grover A, Laski L, Roa M (2018). Accelerate progress—sexual and reproductive health and rights for all: report of the Guttmacher–Lancet Commission. Lancet.

[27] World Health Organization (2010). Packages of interventions for family planning, safe abortion care, maternal, newborn and child health.

[28] Narasimhan M, Allotey P, Hardon A (2019). Self care interventions to advance health and wellbeing: a conceptual framework to inform normative guidance. BMJ.

[29] Inter-agency Working Group on Reproductive Health in Crises. Inter-agency field manual on reproductive health in humanitarian settings 2018: Inter-agency Working Group on Reproductive Health in Crises; 2018. 26203479

[30] Tran NT, Tappis H, Ameyaw EK, Dawson A (2020). Self-care interventions for sexual and reproductive health in humanitarian and fragile settings: scoping review registration. Open science framework.

[31] Keesara S, Jonas A, Schulman K (2020). Covid-19 and health care’s digital revolution. N Engl J Med.

[32] International Telecommunication Union (2019). Yearbook of statistics - telecommunication/ICT indicators - 2009-2018. 2019 edn.

[33] Yankah E, Mohamed O, Wringe A, Afaneh O, Saleh M, Speed O, et al. Feasibility and acceptability of mobile phone platforms to deliver interventions to address gender-based violence among Syrian adolescent girls and young women in Izmir, Turkey. Vulnerable Child Youth Stud. 2019;15(2):133–43.10.1080/17450128.2019.1687965.

[34] The Partnership for Maternal, Newborn & Child Health (2019). Digital opportunities for displaced women, children and adolescents.

[35] World Health Organization (2019). WHO guideline: recommendations on digital interventions for health system strengthening.

[36] Doocy S, Paik KE, Lyles E, Hei Tam H, Fahed Z, Winkler E, Kontunen K, Mkanna A, Burnham G (2017). Guidelines and mHealth to improve quality of hypertension and type 2 diabetes care for vulnerable populations in Lebanon: longitudinal cohort study. JMIR Mhealth Uhealth.

[37] Tran NT, Tappis H, Spilotros N, Krause S, Knaster S (2020). Not a luxury: a call to maintain sexual and reproductive health in humanitarian and fragile settings during the COVID-19 pandemic. Lancet Glob Health.

[38] Schlecht J (2012). Incorporating sexual and reproductive health into emergency preparedness and planning - lessons learned from national-level efforts in Haiti, Uganda and South Sudan.

